# Prognostic impact of programmed cell death-1 (PD-1) and PD-ligand 1 (PD-L1) expression in cancer cells and tumor-infiltrating lymphocytes in ovarian high grade serous carcinoma

**DOI:** 10.18632/oncotarget.6429

**Published:** 2015-11-29

**Authors:** Silvia Darb-Esfahani, Catarina Alisa Kunze, Hagen Kulbe, Jalid Sehouli, Stephan Wienert, Judith Lindner, Jan Budczies, Michael Bockmayr, Manfred Dietel, Carsten Denkert, Ioana Braicu, Korinna Jöhrens

**Affiliations:** ^1^ Institute of Pathology, Charité Universitätsmedizin Berlin, Berlin, Germany; ^2^ Tumorbank Ovarian Cancer Network, Department of Gynecology, Charité Universitätsmedizin Berlin, Berlin, Germany; ^3^ Department of Gynecology, Charité Universitätsmedizin Berlin, Berlin, Germany; ^4^ VM Scope GmbH, Berlin, Germany

**Keywords:** high grade serous carcinoma, ovarian, PD-1, PD-L1, tumor-infiltrating lymphocytes

## Abstract

**Aims:**

Antibodies targeting the checkpoint molecules programmed cell death 1 (PD-1) and its ligand PD-L1 are emerging cancer therapeutics. We systematically investigated PD-1 and PD-L1 expression patterns in the poor-prognosis tumor entity high-grade serous ovarian carcinoma.

**Methods:**

PD-1 and PD-L1 protein expression was determined by immunohistochemistry on tissue microarrays from 215 primary cancers both in cancer cells and in tumor-infiltrating lymphocytes (TILs). mRNA expression was measured by quantitative reverse transcription PCR. An *in silico* validation of mRNA data was performed in The Cancer Genome Atlas (TCGA) dataset.

**Results:**

PD-1 and PD-L1 expression in cancer cells, CD3+, PD-1+, and PD-L1+ TILs densities as well as PD-1 and PD-L1 mRNA levels were positive prognostic factors for progression-free (PFS) and overall survival (OS), with all factors being significant for PFS (*p* < 0.035 each), and most being significant for OS. Most factors also had prognostic value that was independent from age, stage, and residual tumor. Moreover, high PD-1+ TILs as well as PD-L1+ TILs densities added prognostic value to CD3+TILs (PD-1+: *p* = 0.002,; PD-L1+: *p* = 0.002). The significant positive prognostic impact of PD-1 and PD-L1 mRNA expression could be reproduced in the TCGA gene expression datasets (*p* = 0.02 and *p* < 0.0001, respectively).

**Conclusions:**

Despite their reported immune-modulatory function, high PD-1 and PD-L1 levels are indicators of a favorable prognosis in ovarian cancer. Our data indicate that PD-1 and PD-L1 molecules are biologically relevant regulators of the immune response in high-grade serous ovarian carcinoma, which is an argument for the evaluation of immune checkpoint inhibiting drugs in this tumor entity.

## INTRODUCTION

High-grade serous carcinoma (HGSC) is the major histological subtype (approximately 70%) of ovarian carcinomas. It is a poor-prognosis tumor (5-year survival rate 40%), which is due to late diagnosis (75% in FIGO III/IV) as well as the development of resistance to standard platinum-based chemotherapy. However, high numbers of tumor-infiltrating lymphocytes (TILs) have repeatedly been shown to provide a significant survival advantage in ovarian carcinoma. Particularly the presence of T cells (CD3+) and various T cell subpopulations (e.g. CD4+, CD8+, CD103+) are indicators of a better prognosis, [1, 2, 3] strongly suggesting that the anti-tumoral immune response could be exploited as a therapeutic option.

Immune checkpoint inhibitors constitute a novel class of cancer therapeutics that do not target the cancer cell itself but rather ligands and receptors on T cells that attenuate the anti-tumoral immune reaction and increase immune tolerance (for review see [4, 5]). Emerging agents are antibodies targeting the checkpoint molecules programmed cell death 1 (PD-1, PDCD1) and its ligand PD-L1 (B7-H1, CD274). PD-1 is a member of the immunoglobulin superfamily B7 involved in immunomodulation and expressed on the surface of activated T cells especially on germinal center-associated T cells as well as on TILs. The PD-1 pathway has its main function in developing peripheral tolerance.[4, 5] Activation of PD-1 by the two known ligands PD-L1/PD-L2 provoke a suppression of T-cell receptor signaling. Thereby, these two ligands interact with PD-1 on activated T-cells, which induces the inhibition [6] resulting in the down regulation of the immune response during resolution of an infection, during development of self-tolerance, or within the tumor microenvironment. [7]

PD-L1 is induced on monocytes and epithelial cells, upon IFN-gamma stimulation [5], whereas for the up-regulation in dendritic cells other activators are involved. In B-cells PD-L1 is upregulated by surface cross-linking. [8] In tumors PD-L1 up-regulation occurs either by constitutive oncogenic signaling *via* AKT or STAT3, a mechanism termed intrinsic immune resistance, or by IFN-gamma produced by activated T cell or NK cells (adaptive resistance). [4, 5] The micromilieu in cancer is complex and depends on the activation of different immune cell populations and their subpopulations. Our study focused on the expression patterns of PD-1 and PD-L1 in tumor cells as well as in intratumoral T-cells, which we further differentiated into the CD4 and CD8 subpopulation.

PD-1 inhibitors (nivolumab, pembrolizumab) have been approved in therapy-refractory malignant melanoma, and together with PD-L1 inhibitors (MPDL3280A, MEDI4736) are investigated in clinical trials on various recurrent or metastatic malignancies to date. Early clinical trials in ovarian carcinoma are ongoing. [9, 10] Reliable biomarkers predictive of response to PD-1 or PD-L1 targeting drugs have not been fully established to date. As the PD-1 pathway is relevant in the tumor microenvironment, tissue-based markers seem to be the most promising. PD-L1 expression on cancer cells as well as PD-1 expression in TILs is associated with response in some studies, however, their value remains conflictive to date (for review see [11]). Potential other predictive biomarkers might be the mutational burden, which is associated with increased presentation of neo-antigens by tumor cells, [12] or the density of CD8+ TILs in the invasive tumor margin.[13]

To estimate if a tumor entity might constitute a candidate neoplasm for evaluation of a specific cancer therapeutic, the expression pattern of the drug target as well as its clinical relevance are of interest. We therefore systematically evaluated the expression of PD-1 and PD-L1 in a cohort of 215 primary ovarian high-grade serous carcinomas by determining protein expression in cancer cells and TILs as well as mRNA expression.

## RESULTS

### Expression pattern of PD-1 and PD-L1

#### Expression in cancer cells

**Figure 1:**
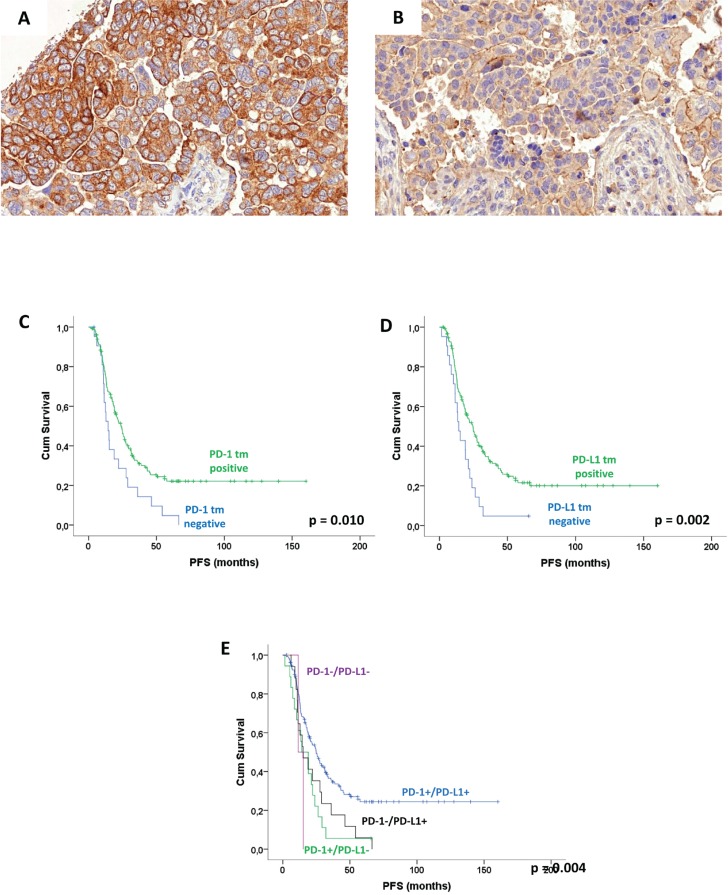
Expression pattern of PD-1 and PD-L1 in high-grade serous ovarian carcinoma cancer cells Membrane-accentuated moderate PD-1 expression in cancer cells **A**. Delicate membranous expression of PD-L1 cancer cells **B**. Kaplan-Meier analysis for membranous PD-1 and PD-L1 expression in cancer cells: PFS according to PD-1 expression **C**., PFS according to PD-L1 expression **D**., PFS according to PD-1/PD-L1 combination **E**. (p: log rank test).

Data on PD-1 and PD-L1 expression in cancer cells were available for *n* = 201 and *n* = 202 cases, respectively. If positive, both markers showed a membrane-accentuated expression, which was also often accompanied by a cytoplasmic expression (Figure 1A, 1B, Suppl. Figure S2). Membranous and cytoplasmic expression (IRS values) were highly correlated with each other (*p* < 0.0001 each). A significant number of cases did not show any membranous expression on cancer cells (IRS = 0; PD-1: *n* = 22, 10.8%, PD-L1: *n* = 24, 11.7%), and for further statistical analyses we decided to split the study group into cases with no PD-1/PD-L1 expression (IRS = 0) and cases with any expression. Membranous PD-1/PD-L1 expression were not correlated to each other (*p* = 1.000, Chi square). As no prognostic effect of cytoplasmic PD-1 or PD-L1 expression in cancer cells could be detected (not shown), only data on membranous expression of these markers will be given subsequently.

#### Expression in TILs

**Figure 2:**
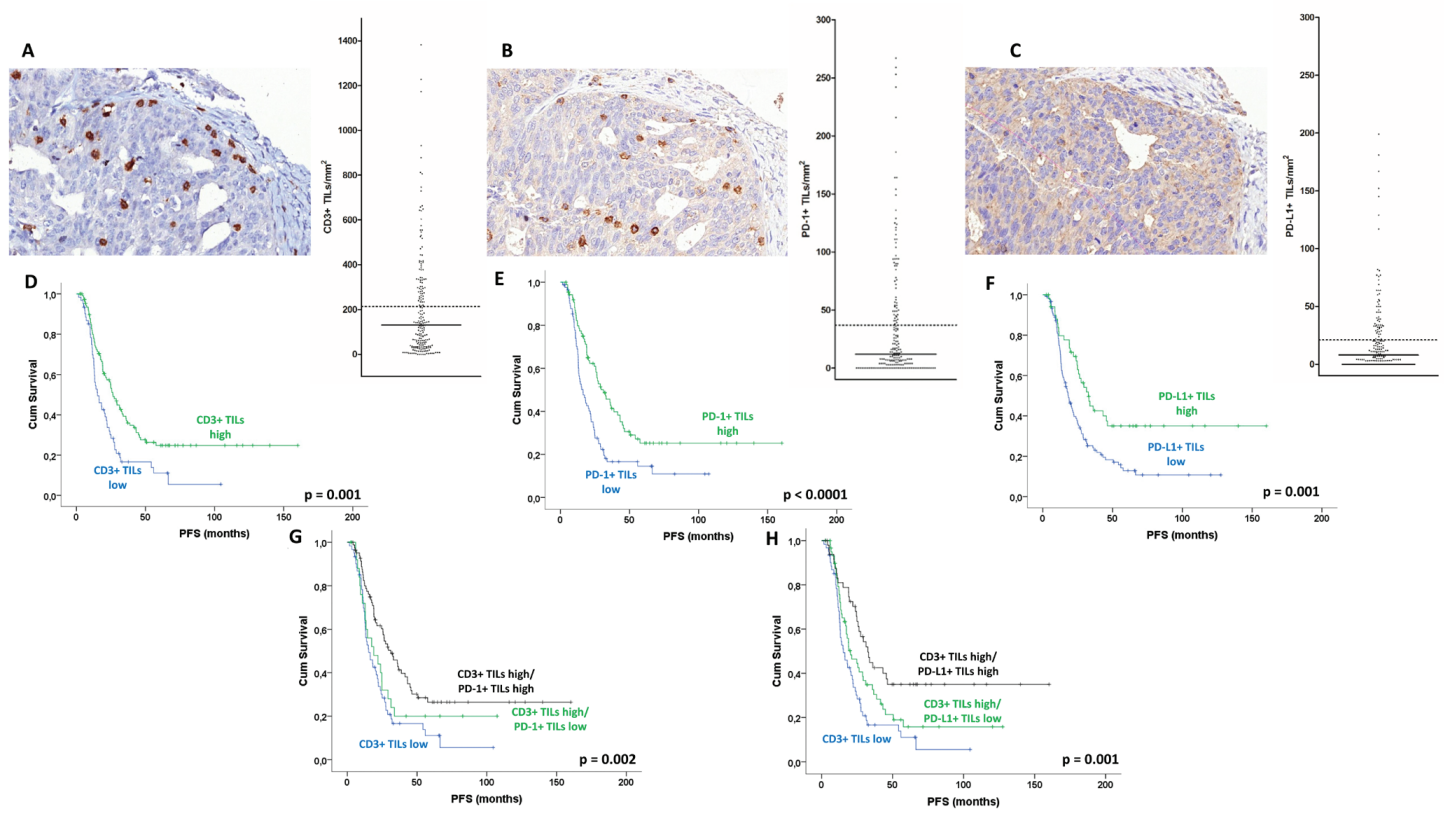
TILs in ovarian high-grade serous carcinoma Intraepithelial CD3+ TILs **A.**, PD-1+ TILs **B.**, and PD-L1+ TILs, an additional faint staining for PD-L1 is seen in tumor cells **C.**. Corresponding tumor areas are shown, the diagrams on the right show the distribution of TILs/mm^2^ tumor area (bars: median, dotted lines: means). Kaplan-Meier analysis for intraepithelial TILs/mm^2^ tumor area: PFS according to CD3+ TILs **D.**. PFS according to PD-1+ TILs **E.**. PFS according to PD-L1+ TILs **F.**. (p: log rank test).

As stromal TILs had a weaker (CD3) or non-significant impact on prognosis (PD-1, PD-L1) as compared to intratumoral TILs (data not shown), data for the latter only are are given subsequently. We separately counted CD3+ as well as PD-1+ and PD-L1+ TILs in identical tumor areas (evaluable cases: *n* = 200 for each marker). All TILs markers showed a positively skewed distribution, which means that the median was lower than the mean: Intraepithelial CD3+ TILs numbers (per 5 HPF) ranged from 0 to 543, however most cases had rather low numbers of CD3+ TILs (median: 34/5 HPF, mean: 65/5 HPF, Figure 2A). Numbers of PD-1+ and PD-L1+ TILs were significantly lower (medians: 3 and 2 per 5 HPF, means: 11/5 HPF and 6/5 HPF, respectively, Figure 2B, 2C). The T cell infiltrate was predominantly composed of CD8+ TILs, as these were significantly more numerous than CD4+ TILs, the positively skewed distribution was seen for those markers, too (Suppl. Figure S3). As the amount of non-tumoral areas (stroma, necrosis) may significantly differ between cases, we calculated the area of tumor cells exclusively for each case and thereby determined the density of TILs (per mm^2^) for each marker. Statistical analyses are shown for these values subsequently. CD3+, CD4+, CD8+, PD-1+, and PD-L1+ TILs/mm^2^ highly correlated with each other (Spearman's rho: 0.495-0.777, *p* < 0001 for each test). PD-1 expression in cancer cells showed a borderline positive correlation with CD3+ TILs/mm^2^ (*p* = 0.05, Mann-Whitney), but not with PD-1+, PD-L1+, CD4+, or CD8+ TILs, and PD-L1 expression in cancer was not significantly associated with TILs at all (*p* > 0.05 each).

**Table 1:** Characteristics of the study group

	n (%)
**total**	215 (100%)
**age**	
<=60 years	108 (50.2)
>60 years	107 (49.8)
**FIGO stage**	
FIGO I	16 (7.4)
FIGO II	13 (6.0)
FIGO III	162 (75.3)
FIGO IV	24 (11.2)
**residual tumor *(FIGO II-IV, n=199)***	
no	110 (63.9)
yes	62 (36.0)
*missing*	27 *(13.5)*
**chemotherapy**	
platinum-based	163 (95.9)
other	5 (2.9)
none	2 (1.2)*
*missing*	*45 (20.9)*

* FIGO I

#### Interaction between PD-1 and PD-L1 expression and CD4+ and CD8+ TILs density

PD-1 and PD-L1 cancer cell and TILs expressions were further investigated as to their relationship to the CD4+ and CD8+ T cell population when we grouped cancer cell expression in four groups. We did not observe a dependence of CD4+ or CD8+ TILs density and expression of PD-1 or PD-L1 in cancer cells (Suppl. Figure 4A, 4B; we omitted PD-1-/PD-L1- tumors form this analysis, as sample size was quite small here (*n* = 2)). We furthermore investigated the potential dependence of CD4+ and CD8+ TILs density from combined cancer cell and TILs expression of PD-1 and PD-L1. As shown in Suppl. Figure 4C, 4D, numbers of CD4+ and CD8+ TILs were correlated with PD-1+ and PD-L1 TILs status and decreased from double positive cases (PD-1+ TILs+/PD-L1 TILs+), over cases with only one high TILs marker (PD-1+ TILs+ or PD-L1+), to cases with both low TILs markers (PD-1+ TILs-/ PD-L1+ TILs-). However again, this was same for tumors with either one or both markers positive in cancer cells. Taken together, our data indicate that the CD4+ and CD8+ T cell infiltrate, similarly to PD-1+ and PD-L1+ T cells is not regulated by PD-1 or PD-L1 expression in cancer cells but rather is proportional to the amount of T cell infiltration in general.

#### mRNA expression

**Figure 3:**
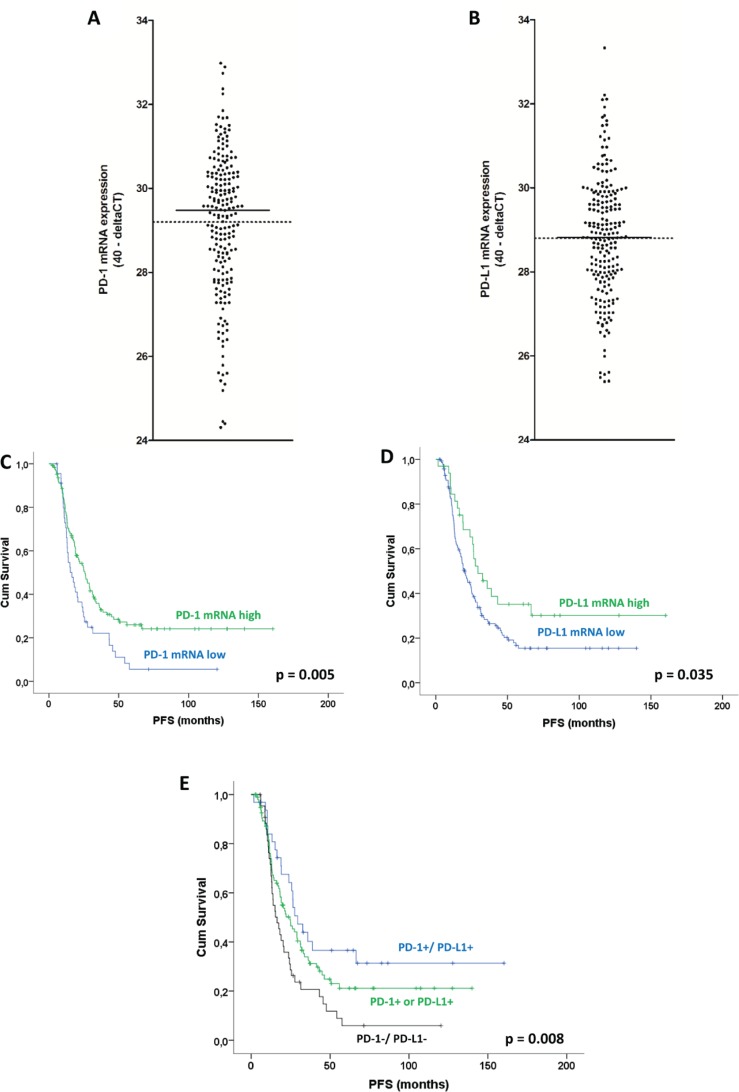
PD-1 and PD-L1 mRNA expression in ovarian high-grade serous carcinoma Distribution of PD-1 **A.** and PD-L1 **B.** mRNA expression levels in the study group (bars: median, dotted lines: means). Kaplan-Meier analysis for PD-1 and PD-L1 mRNA expression: PFS **C.** according to PD-1 mRNA expression. PFS **D.** according to PD-L1 mRNA expression. PFS **E.** according to PD-1/PD-L1 combination (cases with only one positive marker were grouped together as only one tumor was PD-1-/PD-L1+). (p: log rank test).

Informative mRNA expression data were available for 200 cases for PD-1 and 204 cases for PD-L1. Both markers showed a rather low expression with PD-1 40-deltaCT values ranging from 24.31 to 32.98 (median 29.49) and PD-L1 data ranging from 25.39 to 33.33 40-deltaCT (median 28.83; Figure 3A, 3B). Both PD-1 and PD-L1 mRNA expression were significantly correlated with each other (Spearman rho 0.627, *p* < 0.0001), as well as with CD3+, PD-1+, and PD-L1+ TILs (Spearman rho between 0.227 and 0.477, all *p* < 0.004). There was a trend towards a positive correlation between PD-1 mRNA and PD-1 expression in cancer cells (*p* = 0.082, Mann-Whitney), however not between PD-1 mRNA and PD-L1 expression in cancer cells or between PD-L1 mRNA and PD-1/PD-L1 expression in cancer cells (*p* > 0.1 each).

### Prognostic effect of PD-1 and PD-L1 expression

#### Prognosis according to expression in cancer cells

Both PD-1 and PD-L1 expressions in cancer cells were significantly linked to a better PFS (PD-1: *p* = 0.010, PD-L1: 0.002, Figure 1C, 1D, Table 2). PD-L1 expression has also significant impact on OS (*p* = 0.045), while for PD-1 expression only a trend was seen for OS (*p* = 0.059, Suppl. Table S1). The significant prognostic impact of PD-1 and PD-L1 expression in cancer cells was retained in multivariate analysis, and was independent from patient age, FIGO stage, and residual tumor after surgery (*p* < 0.05, Table 2, Suppl. Table S1). (Further significant prognostic factors in our cohort were age, FIGO stage, and residual tumor for OS and FIGO stage for PFS, not shown). Interestingly, the combination of PD-1 and PD-L1 staining indicated a dose-effect on survival, as in PD-1+/PD-L1+ double positive tumors PFS (*p* = 0.004) and OS (*p* = 0.051) were longest, in cases with only one positive marker were intermediate, and in double negative cases were worst, however as only two tumors were PD-1-/PD-L1- the data for the latter group must be interpreted with caution (Figure 1E, Table 2, Suppl. Table S1). We also investigated whether cutoffs points in the high range of PD-1 or PD-L1 expression using IRS values and percentage groups were prognostic, too, however the most significant effect was found for the cutoff described above (no *vs* any expression; not shown).

**Table 2:** Survival Analysis: Progression-Free Survival

	*univariate*			*multivariate*	
	no of events/ no of cases	median survival, months (SE)	p (log rank)	HR (95% CI)	p
**PD-1 in tumor cells (membranous)**					
Negative	20/ 21	14.46 (2.0)		1	
positive	104/ 151	23.85 (2.2)	0.010	0.57 (0.34-0.95)	0.032
**PD-L1 in tumor cells (membranous)**					
Negative	18/ 24	14.13 (1.7)		1	
positive	106 / 153	24.61 (2.5)	0.002	0.41 (0.23-0.71)	0.002
**PD-1/PD-L1 in tumor cells combination**					
PD-1+/PD-L1+	88/ 134	24.84 (2.9)		1	
PD-1+/PD-L1-	17/ 18	14.13 (6.0)		2.64 (1.48-4.68)	
PD-1-/PD-L1+	17/ 18	15.11 (4.2)		1.78 (1.02-3.10)	
PD-1-/PD-L1-	2/ 2	11.53 (−)	0.004	1.92 (0.26-14.37)	0.004
**CD3+ TILs/mm^2^**					
<=65	51/ 61	15.31 (2.7)		1	
> 65	73/ 111	26.45 (2.3)	0.001	0.65 (0.43-0.98)	0.041
**PD-1+ TILs/mm^2^**					
<=11	68 / 84	16.36 (2.3)		1	
>11	56 / 88	31.11 (4.7)	<0.0001	0.54 (0.36-082)	0.003
**PD-L1+ TILs/mm^2^**					
<=20	94 / 120	18.33 (1.7)		1	
>20	30 / 52	32.59 (4.0)	0.001	0.63 (0.40-1.01)	0.053
**PD-1/CD3 TILs combination**					
CD3+ TILs low	51/61	15.31 (2.7)		1	
CD3+ TILs high/PD-1+ TILs low	20/27	19.00 (6.2)		1.95 (0.57-1.94)	
CD3+ TILs high/PD-1+ TILs high	53/84	31.11 (4.7)	0.002	0.57 [0.37-0.90)	0.022
**PD-L1/CD3 TILs combination**					
CD3+ TILs low	51/61	15.13 (2.7)		1	
CD3+ TILs high/PD-L1+ TILs low	44/61	20.3 (3.9)		0.74 (0.47-1.17)	
CD3+ TILs high/PD-L1+ TILs high	29/50	32.6 (4.0)	0.001	0.54 (0.32-0.91)	0.069
**PD-1 mRNA (40 – deltaCT)**					
<=28.18	39/45	15.31 (2.3)		1	
>28.18	85/128	25.69 (3.1)	0.005	0.49 (0.32-0.74)	0.001
**PD-L1 mRNA**					
<=30.45	105/142	20.24 (1.7)		1	
>30.45	21/33	29.44 (6.3)	0.035	0.41 (0.23-0.71)	0.001
**PD-1/PD-L1 mRNA combination**					
PD1+/PD-L1+	20/32	29.44 (4.2)		1	
PD1+ or PD-L1+	66/97	24.61 (3.7)		2.14 (1.18-3.88)	
PD1-/PD-L1-	37/43	15.31 (2.0)	0.008	3.60 (1.91-6.83)	<0.0001

SE: standard error; HR: hazard ratio; CI: confidence interval; for each marker multivariate analysis was performed including age (<=60 vs > 60 years), stage (FIGO I/II vs III/IV), and residual tumor (none vs any)

#### Prognosis according to expression in TILs

First we investigated the prognostic impact of TILs using a cutoff-free approach. Due to the fact that TILs distribution was positively skewed, we logarithmized the data and performed Cox regression analysis using continuous data. A high density of CD3+ intratumoral TILs was a significant positive prognostic marker for PFS and OS on the continuous scale: PFS: HR = 0.65 (95% CI 0.49-0.85), *p* = 0.002; OS: HR = 0.64 (95% CI 0.48-0.85), *p* = 0.002. This significant positive impact on prognosis was also seen for PD-L1+ TILs density for both PFS (HR = 0.75 (95% CI 0.58-0.97), *p* = 0.026) and OS (HR = 0.72 (95% CI 0.55-0.96), *p* = 0.025), as well as for PD-1+ TILs density for PFS (HR = 0.78 (95% CI 0.62-0.99), *p* = 0.044), with a lack of significance for OS (HR = 0.80 (95% CI 0.61-1.05, *p* = 0.105).

To determine which amount of TILs infiltration might have the most prominent prognostic impact in the study cohort, we used the online tool Cutoff Finder,[17] which with hazard ratios (univariate Cox regression) could be plotted against each possible cutoff point for each marker. For this analysis, non-logarithmized data were used. Reflecting our finding of a continuous impact of increasing TILs density on prognosis, the resulting charts (Suppl. Figure S5A-S5C) demonstrated that a large range of cutoff points yielded significant results for CD3+, PD-1, and PD-L1 TILs density: Thus, for CD3+ TILs the vast majority of potential cutoff points yielded significant results in Kaplan-Meier analysis, namely 95 out of 125 (75%). Also PD-1+ TILs (29 out of 48 cutoff points, 60.4%), and PD-L1+ TILs (31 out of 61 cutoff points, 49.2%) were robust prognostic markers. The optimal cutoff points for CD3+, PD-1+ and PD-L1+ TILs, which yielded the most significant split of the cohort within the large range of significant cutoff points, were 65, 11, and 20 TILs/mm^2^, respectively. Using this optimal cutoff, Kaplan-Meier analysis showed that CD3+ TILs had a significantly favorable impact on PFS and OS (*p* = 0.001, *p* = 0.003), with borderline significance in multivariate analysis (Figure 2D; Table 1, Suppl. Table S1). A significantly better prognosis was also seen tumors with a high PD-1+ or PD-L1+ TILs density, but only PD-1+ TILs showed independent prognostic impact on PFS and OS (Figure 2E, 2F, Table 2, Suppl. Table S1).

#### Prognostic interaction of CD3+ and PD-1+/PD-L1+ TILs density

As all TIL subpopulations in our study were highly correlated with each other, we wondered if the prognostic effect of PD-1+ and PD-L1+ TILs might only be a bystander effect of the impact of T cell infiltration by itself. We therefore investigated the prognostic effect of combined CD3+ and PD-1+/PD-L1+ TILs density. Surprisingly, tumors that had a high PD-1+ TILs or PD-L1 TILs density in addition to high CD3+ TILs had a better prognosis (both PFS and OS) than tumors with low PD-1+ or PD-L1+ TILs counts despite of a high CD3 infiltration (Figure 2G, 2H, Table 2, Suppl. Table S1). Thus, PD-1+ or PD-L1+ TILs added prognostic information to CD3+ TILs. In a bi-variate Cox regression analysis including either CD3+ TILs and PD-1+ TILs or CD3+ TILs and PD-L1+ TILs, PD-1+ TILs and PD-L1+ TILs retained their impact on PFS, which was independent from CD3+ TILs density (PD-1+ TILs: HR = 0.55, 95% CI = 0.31-0.95, *p* = 0.032; PD-L1+ TILs: HR = 0.60, 95% CI = 0.38-0.94, *p* = 0.027).

#### Prognosis according to mRNA expression

Investigating PD-1 and PD-L1 mRNA expression in a cutoff-free approach, we found that PD-1 mRNA expression was a positive prognostic factor on the continuous scale (HR = 0.88, 95% CI 0.080-0.97 per 40 - deltaCT, *p* = 0.012), but PD-L1 mRNA was not (*p* = 0.609). Significant cutoff points - similarly to TILs density - were found over a rather large range: for PD-1 mRNA 39 out of 128 cutoff points (39.5%), and for PD-L1 mRNA 49 out of 128 cutoff points (38.3%) showed significant positive prognostic impact (Suppl. Figure S5D, S5E). Using the most significant cutoff point at 28.18 or 29.99, respectively, both markers were significant positive prognostic factors for PFS (*p* = 0.005 and *p* = 0.035, Figure 3C, 3D, Table 2) and OS (0.036 and *p* = 0.045, Suppl. Table S1). Significance for PD-1 and PD-L1 mRNA expression was retained in multivariate analysis for PFS (*p* = 0.001 and *p* = 0.001, Table 2), and OS (*p* = 0.042 and *p* = 0.047, Suppl. Table S1). Similarly to protein expression of cancer cells, the combination of mRNA expression of both markers showed a dose-effect on survival with tumors positive for both markers showing the longest, tumors with only one positive marker and intermediate, and tumors negative for both markers showing the shortest survival time (PFS: *p* = 0.008, OS: *p* = 0.045; Figure 3E, 3F, Table 2, Suppl. Table S1).

#### *In silico* validation of mRNA expression in the TCGA datasets

**Figure 4:**
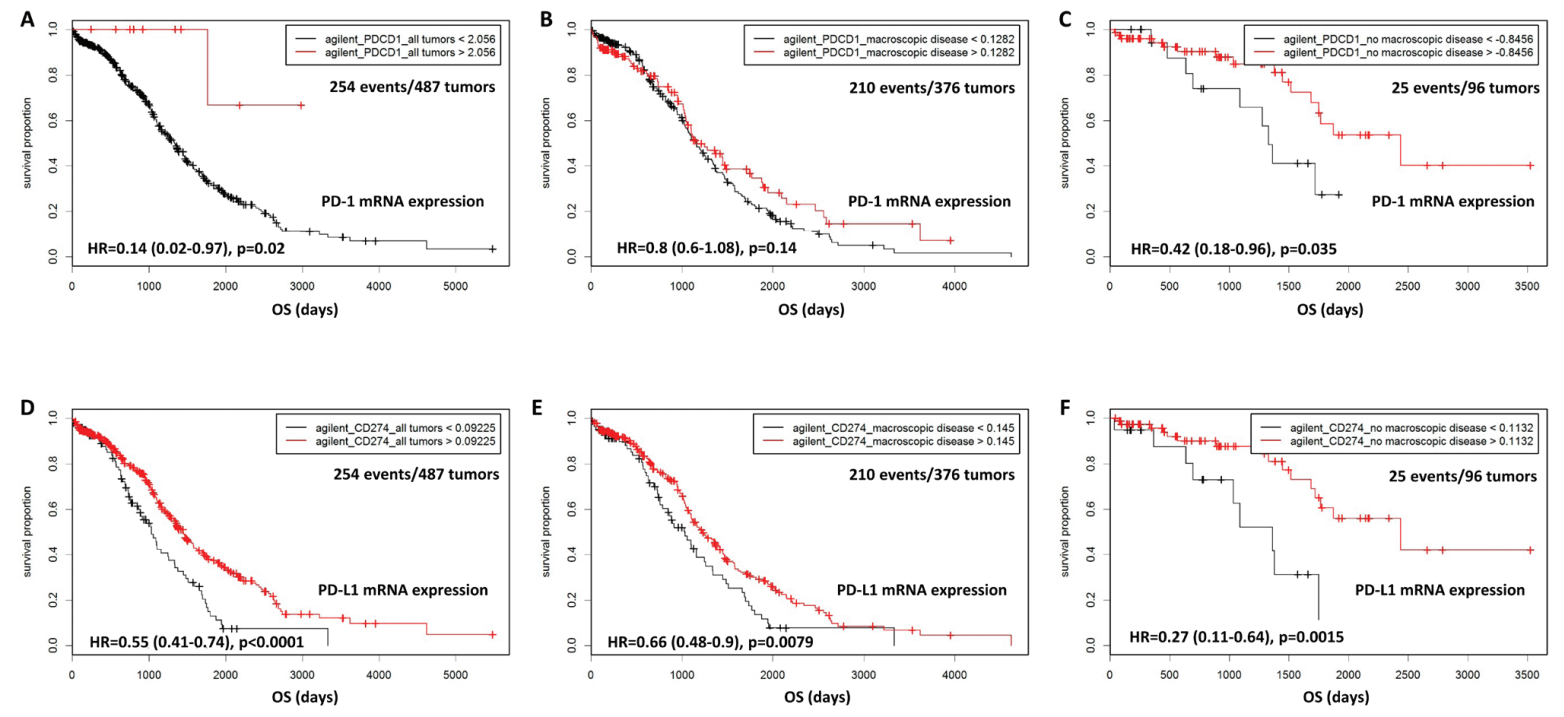
Validation of mRNA data in the TCGA cohort of primary high-grade serous carcinomas OS according to PD-1 (syn PDCD1) expression in the total cohort **A.**, in the group of cancers that had been operated with residual tumors **B.**, and in the group without residual tumor after surgery **C.**. OS according to PD-L1 (syn. CD274) expression in the total cohort **D.**, in the group of cancers that had been operated with residual tumors **E.**, and in the group without residual tumor after surgery **F.**

Gene expression datasets form The Cancer Genome Atlas project on primary high-grade serous ovarian carcinomas [18] were analyzed for the prognostic impact of PD-1 and PD-L1 mRNA as to OS in an independent cohort. Data for PD-1 expression were available for three platforms (Affymetrix, Agilent, RNAseq) and for PD-L1 expression for two platforms (Agilent, RNAseq). Kaplan-Meier plots are shown representatively for Agilent data in Figure 4: PD-L1 expression (syn. CD274 in TCGA) was a robust positive prognostic factor in the total study cohort (Agilent: 113 out of 444 cutoffs significant (25.5%); optimal cutoff *p* < 0.0001, Figure 4D; RNAseq: 117 out of 380 cutoffs significant (30.8%), optimal cutoff: *p* < 0.0001, not shown) as well as in the subgroup with residual tumor after surgery (Agilent: *p* = 0.0079, Figure 4E; RNAseq: *p* = 0.0012, not shown), and furthermore in cancers without residual tumor after surgery (Agilent: *p* = 0.0015, Figure 4F; trend for RNAseq: *p* = 0.11, not shown). PD-1 expression (syn. PDCD1 in TCGA) was also a positive prognostic factor for the total cohort, however its prognostic value was of reduced robustness as only few cutoffs were significant: Agilent: 14 out of 460 cutoffs significant (3.0%), optimal cutoff *p* = 0.02, Figure 4A), which was also seen in Affymetrix data (36 out of 445 cutoffs significant (7.9%), optimal cutoff *p* = 0.013, not shown), however missed significance in RNA seq data (*p* = 0.065, not shown). In the subgroup of tumors with residual tumor after surgery a trend for better OS was seen for PD-1 expression (Agilent: *p* = 0.14, Figure 4B; Affymetrix: *p* = 0.11, not shown, significant for RNAseq: *p* = 0.036, not shown). A partially significant effect was also seen in the subgroup without residual tumor after surgery (Agilent: *p* = 0.035, Figure 4C; Affymetrix: *p* = 0.11, not shown; RNAseq: *p* = 0.19, not shown).

#### Prognostic effect in clinical subgroups

We additionally investigated the prognostic value of PD-1 and PD-L1 expression only for patients that had documented platinum-based chemotherapy. PD-1 and PD-L1 expression, both in cancer cells and TILs as well as mRNA expression were significant prognostic factors for PFS in this well-defined subgroup, too, and significance was retained for all but PD-L1+ TILs and PD-L1/CD3 TILs combination in multivariate analysis (Suppl. Table S2). Data for OS showed similar significances (not shown). As residual tumor after surgery is the most important established prognostic marker for ovarian carcinoma, the performance of prognostic markers in the subgroups of patients with or without residual tumor is of clinical interest. We therefore performed survival analyses stratified for residual tumor (FIGO stages II-IV) in patients with platinum-based chemotherapy. As shown in Suppl. Table S3, in patients without residual tumor all markers were significant for PFS, with only PD-L1+ TILs missing significance in multivariate analysis including age (< = 60 *vs* > 60 years) and stage (II *vs* III/IV). Similar results were obtained for OS (not shown).

## DISCUSSION

In this study, we systematically investigated PD-1 and PD-L1 expression in primary high-grade serous ovarian carcinoma. High expression in cancer cells and mRNA expression were favorable prognostic factors, as was the density of PD-1+ and PD-L1+ TILs, moreover, this favorable prognostic effect of TILs was independent from the T cell infiltrate in general. We validated our findings on the mRNA level in the independent cohort of high-grade serous carcinomas from TCGA.

Our findings are in line with a recent report on a positive prognostic effect of PD-1+ TILs in a cohort of 195 high-grade serous carcinomas. [14] This study found quite similar median numbers of PD-1+ TILs in high-grade serous ovarian carcinomas: approximately *n* = 12 PD-1+ TILs/mm^2^ (tumor and stroma not separated), our study: *n* = 12 PD-1+ TILs/mm^2^ (intratumoral only), and it is remarkable that this small subpopulation of T cells obviously has a biological significance as measured by patient prognosis. This rather surprising finding prompted us to investigate whether the positive prognostic effect of PD-1+ and PD-L1 TILs might be a bystander effect of the intratumoral T cell infiltration in general. The absolute counts of PD-1 and PD-L1 TILs in high-grade serous carcinoma were strongly associated with CD3+ TILs, but only a small fraction of T cells actually expressed PD-1 and PD-L1 and their amount seemed to increase proportionally to the intratumoral immune reaction - as reflected by the density of the lymphocyte infiltrate - potentially in terms of a feedback activation. We have made similar observations in breast cancer, where we found that mRNA levels of a plethora of immune-related genes, PD-1 and PD-L1 included, were positively correlated with each other as well as with the density of TILs, irrespective of their immune-promoting or immune-inhibitory function, and were all strong indicators of a higher response rate to neoadjuvant chemotherapy. [16] However, our analyses in ovarian cancer rather suggest that the positive prognostic implication of PD-1+ and PD-L1+ TILs is independent from the amount of T cells and not only a bystander effect of an activated immune response. The available data on this topic are conflictive to date, and both favorable and unfavorable prognostic effects of PD-1+ or PD-L1+ TILs in human carcinomas have been reported. [15, 16, 17, 18] The complex interaction of immune effector cells within the tumor microenvironment very likely impacts the biological significance of particular immune markers and seems to be dependent on tumor entity. It is well known that the micromilieu of tumors has a broad complexity. [19] Subpopulations of CD4+ T cells for instance can have anti-tumorigenic effects when the Th1 subset is active in secreting pro-inflammatory cytokines, whereas a pro- tumorigenic effect can be predominant when Th2 cells secrete anti-inflammatory cytokines. The effect of T regulatory (Treg) cells obviously depends on the interaction with distinct immune cell subpopulations. The CD8+ T-cell population attacks the cancer cells by producing cytotoxic molecules like perforin. Other T-cell subtypes are of prognostic relevance, too. Treg cells [20] as well the circulating Th17 cells [21] play an important role in orchestrating the behavior of different tumor entities. Until now the structure of this network and the importance of the different players are not well understood. Further investigations regarding the different T-cell compartments are needed to integrate our novel and surprising results.

An impact of PD-L1 on γδ T cells has been reported. One study investigated the expression and function of PD-1 in human γδ T cells that recognize phosphoantigens. [22] The results of this study suggested that TCR triggering may partially overcome the inhibitory effect of PD-1 in γδ T cells. Iwamura et al. described that siRNA-mediated knockdown of PD-L1 or -L2 enhanced the IFN-gamma production and antigen-specific cytotoxicity of αβ cells and peripheral blood mononuclear cells transduced with a retroviral vector encoding MAGE-A4-specific T-cell receptor αβ chains and also increased their effector functions by this modification. [23] We would therefore expect a change of the proportion of αβ cells *vs* γδ cells, an issue, which would be worth further investigations.

The localization of TILs seems to be of major relevance as to their prognostic impact. Our data showing a consistent positive impact of intratumoral (= intraepithelial) TILs on survival is in line with several previous reports. [2, 3, 24, 25] However stromal lymphocytes, which can be quite numerous in certain tumors, are very likely to play an important role in the anti-tumoral immune response, too, although their significance for prediction of survival is lower than the one of intratumoral TILs. The role and potential interaction of intratumoral and stromal TILs in ovarian cancer is a highly interesting research topic beyond the scope of our present paper.

Currently, the expression of PD-L1 on tumor cells is regarded as an immune-escape mechanism of the tumor, as it attracts PD-1 expressing immune-inhibitory TILs. However, this mechanism is expected to rather result into a negative impact of tumorcellular PD-L1 expression on survival, and this is reported, e.g. for breast cancer, NSCLC, renal cell carcinoma (for meta-analysis see [26]), osteosarcoma, [27] or advanced melanoma. [28] Of note, our study is not the only one to describe a favorable prognostic impact of PD-L1 expression in cancer cells. E.g. Schmidt et al. investigated FFPE tissue of 321 patients with NSCLC and could demonstrate that PD-L1 cancer cell expression is a prognostic factor for NSCLC patients with the squamous cell subtype showing a better outcome. [29] Also Kluger et al. described a better overall survival in patients with malignant melanomas associated with high levels of PD-L1 expression. [30] An older study actually showed a negative prognostic impact of PD-L1 expression in ovarian cancer cells, however, this study investigated various (molecularly and clinically quite different) histological types together and only 40% of the cohort consisted of serous carcinomas, therefore those results are hard to compare with ours. [31]

Today it can only be speculated why components of the PD-1 pathway are in some instances (such as in ovarian carcinoma) linked to a favorable prognosis. A positive impact of PD-L1 expression of tumor cells as seen in our study might be explained by a compensatory up-regulation of this marker in a microenvironment that threatens the tumor by an active immune response. An association between PD-L1 on tumor cells and a high TILs density would be an argument for this hypothesis, and has as well been described by Hamanishi et al. in their study on various ovarian cancer histotypes [36] as well as in breast cancer [32, 33] however this hypothesis remains speculative to date. We hypothesize that also the positive prognostic impact of PD-1+ and PD-L1+ TILs is based on regulatory and not yet completely elucidated mechanisms within the immune network in the tumor microenvironment as outlined above. Thus, regulatory and immune-suppressive T cells might be up-regulated during an enhanced anti-tumoral immune response.

The fact that PD-1 and PD-L1 are expressed in two tumoral compartments (cancer cells and TILs) and that this expression has prognostic impact strongly suggest that the PD-1/PD-L1 axis has a biological relevance in high-grade serous ovarian carcinoma. This entity therefore appears as a candidate malignancy in which PD-1/PD-L1 targeting drugs should be tested. Although these agents have shown remarkable effects in subsets of patients with in poor-prognosis and therapy-resistant cancers such as NSCLC [34] and renal cell carcinoma, [35] there is still a debate on which biomarkers are most suitable to predict response. A clear predictive effect of PD-L1 expression in cancer has not determined yet, and might be complicated by the fact that antibodies, evaluation methods and cutoff points for determining positivity vary between studies. The difficulty in comparing results from methodologically different studies is illustrated by the fact that we observed PD-1 expression in cancer cells by the use of a particular, carefully validated antibody, while using another antibody, which produced quite similar staining results for TILs, PD-1 cancer cell expression was not seen. Of note, PD-1 expression in cancer cells was described in a recent report on non-small cell lung cancer. [36] Efforts to standardize the interpretation of PD-L1 immunohistochemical stainings in cancer cells and also in TILs are undertaken in several countries to date, e.g. in the ring trial preparation of the German Pathologist's Societies, yet an established interpretation method for PD-L1 (and PD-1) expression is not available yet. In our study the cutoff point with maximal prognostic capacity was in the low expression range (none *vs* any expression), although we are aware of the fact that the cutoff point for response prediction might be different. A recent phase I study on pembrolizumab in NSCLC for the first time validated a previously defined cutoff point of 50% for response prediction, however responses were also seen among patients with tumors with expression below the cutoff indicating that tumoral PD-L1 expression might not constitute the definite predictive marker. [37] For the PD-L1-targeting antibody MPDL3280A a high predictive impact of PD-L1 expression on tumor-infiltrating immune cells was seen in a phase I study on metastatic bladder cancer [38] as well as in a phase I study including multiple advanced or metastatic cancer types, predominantly NSCLC (ovarian carcinoma, *n* = 1). [39] Numbers of PD-L1+ TILs were highly predictive of response, in contrast to the PD-L1 status in cancer cells. In studies on immune checkpoint inhibitors in ovarian carcinoma, a stratification as to tumorcellular and immune-cell-related PD-1/PD-L1 expression should reveal whether PD-1 and PD-L1 apart from their prognostic value are suitable as predictive markers, too. PD-1/PD-L1 mRNA expression was also a robust measure of prognosis in our study, therefore an evaluation of its predictive value is worthwhile.

Our study has several strengths and weaknesses: We only investigated primary tumors, but the current use of immune checkpoint inhibitors is focused on metastatic or recurrent tumors to date. However, the data of Powels et al. indicate that the immunological microenvironment of a tumor might be temporally stable in terms of its predictive value. [43] A comparative study on the immune infiltrate in primary and recurrent high-grade serous carcinomas is ongoing in our lab. Not all cases in our study group had available data on adjuvant chemotherapy. A survival analysis including only patients with documented platinum-based chemotherapy revealed results with significance that was quite similar to the total study groups. Strengths of our study were the rather large sample size, the highly standardized method to quantify TILs, and the in silico validation of our data in the TCGA gene expression datasets.

To summarize, we report a significant prognostic impact of PD-1 and PD-L1 expression in primary high-grade serous ovarian carcinoma. PD-1+ and PD-L1+ TILs increase proportionally to the general lymphocytic infiltrate, however carry independent prognostic information. PD-1 and PD-L1 expression in cancer cells as well as mRNA expression are favorable prognostic markers. Our data indicate that PD-1 and PD-L1 molecules are biologically relevant regulators of immune response in high-grade serous ovarian carcinoma, and that the evaluation of immune checkpoint-inhibiting drugs might be of value in this poor-prognosis cancer type, for which only limited options for targeted therapy are available to date.

## MATERIALS AND METHODS

### Study population

Formalin-fixed and paraffin-embedded (FFPE) surgical specimens from 215 patients with primary ovarian high-grade serous carcinoma were used (Table 1). Most patients (*n* = 165) patients had received surgery in the Department of Gynecology of the Charité and had been included into the TOC project (Tumorbank Ovarian Cancer, www.toc-network.de). Scientific use of TOC and non-TOC cases has been approved by the ethics committee of the Charité. Data on residual tumor mass after surgery (applies for stage II-IV) were available for 152 patients (76.4%). The majority of patients had been treated with standard adjuvant platinum-based chemotherapy (*n* = 163/170, 95.9%). Data on overall survival (OS) were available for all patients. Median OS was 37.9 months, 113 patients died during follow-up (52.6%). Data on progression-free survival (PFS) were available for 185 patients (86.0%; median PFS 19.6 months).

### Immunohistochemistry

Staining was performed on tissue microarrays (TMAs) with two cores for each case, according to standard procedures. In brief, a mouse monoclonal antibody against PD-1 (clone MRQ-22, Zytomed Systems GmbH, Berlin, Germany) was used in a dilution of 1:50. For PD-L1 detection, a rabbit monoclonal antibody was used in a dilution of 1:300 (clone EPR1161(2), Abcam plc., Cambridge, UK). PD-1 and PD-L1 staining was performed using the BondMax™ device (Leica Biosystems GmbH, Wetzlar, Germany). Antigen retrieval and visualisation of bound antibodies were performed employing the manufacturer's protocols and reagents (Bond Polymer Refine, DAB; Leica). CD3 staining was performed using a rabbit polyclonal antibody in a dilution of 1:100 (Dako, Glostrup, Denmark), CD4 was stained with a mouse monoclonal antibody in a 1:20 dilution (clone 1F6, Novocasta/Leica) and CD8 was detected with a mouse monoclonal antibody (1:25, clone C8/144B, Dako), using the BenchMark XT device (Ventana Medical Systems, Inc., Tucson, AZ, USA) and 3,3′-diaminoenzidine peroxide substrate (DAB^+^), as a chromogen. CD3, PD-1, and PD-L1 were stained on consecutive TMA sections enabling the evaluation of corresponding tumor areas. Specificity of the PD-1 and PD-L1 antibodies had been validated before by evaluation of the staining patterns in normal lymphoid tissue and lymphoproliferative diseases, for which a PD-1 or PD-L1 expression had been described (lymphocyte predominant Hodgkin lymphoma, EBV-associated diffuse large B cell lymphoma, classical Hodgkin lymphoma). Stained slides were scanned and evaluated on screen by an experienced pathologist (CAK); doubtful cases were discussed with a senior hematopathologist (KJ) until consensus was achieved.

#### Interpretation of PD-1 and PD-L1 expression in cancer cells

The VM Slide Explorer and VM TMA Evaluator software was used (VMscope GmbH, Berlin, Germany). Intensity and rate of stained cells were determined by two pathologists (CAK in support of KJ) on screen for both membranous and cytoplasmic staining and were combined to a semi-quantitative immuno-reactivity score (IRS). [40] The scoring system is shown as a table in Suppl. Figure S1A.

#### Assessment of TILs

CD3+ TILs were evaluated first: 5 tumor areas in a 400x magnification (high power fields (HPF)) were screen-shotted with the use of VM Slide Explorer 2.2 (VMscope) considering preferentially areas with higher intratumoral TILs density. Microphotographs were subsequently evaluated using the ROI Manager software (CognitionMaster) [41]. In each microphotograph (= HPF) the non-tumor areas (e.g. stroma, necrosis) were labelled to separate them from areas of pure tumor. CD3+ TILs were then separately marked for their location within the tumor epithelium (in direct contact with tumor cells = intratumoral TILs) or within the stroma (= stromal TILs). Lymphocytes in non-epithelial, non-stromal location (e.g. in vessels or in necrosis) were not evaluated. The number of TILs calculated by ROI Manager for each microphotograph were added to obtain TILs/5 HPF. Using the pure-tumor area calculated by ROI Manager for each case, the density of CD3+ intratumoral TILs (per mm^2^) could be assessed. For PD-1+ and PD-L1+ TILs the same tumor areas as for CD3+ TILs were evaluated accordingly. A screen-shot of CD3 evaluation in a representative case is shown in Suppl. Figure S1B. CD4+ and CD8+ TILs were assessed in the same way as CD3+ TILs. Selection of HPFs, labelling of areas as well of TILs was performed by a pathologist (CAK in support of KJ).

#### Comparison of PD-1 and PD-L1 immunohistochemical expression in TMAs and large sections

10 cases with high and low TILs density, respectively, were stained on paired TMAs and large sections. Large sections were stained and evaluated in exactly the same way as TMA spots had been treated before. An exception was PD-1, for which another antibody was used (rabbit monoclonal, clone EP239, Epitomics, Burlingame, CA, USA) because the antibody we used before was not commercially available any more. There was a strong correlation between paired TMA and large section data for CD3+ (Spearman's correlation coefficient 0.771, *p* < 0.0001), PD-L1+ (Spearman's correlation coefficient 0.816, *p* < 0.0001), as well as for PD-1+ TILs density (Spearman's correlation coefficient 0.908, *p* < 0.0001). Membranous PD-L1 expression in tumor cells (IRS values) also correlated in TMA and large sections (Spearman's correlation coefficient 0.578, *p* = 0.008). In the group of cases that were scored as positive on large sections (= any staining, IRS1-12, *n* = 17) all had been classified as positive on TMAs, too. Two of the three cases that were scored as negative on large sections (= no staining, IRS0) had been scored positive on TMA, one with an IRS of 2, the second with an IRS of 3, which both are in the low-expression range. Using the novel PD-1 antibody in large sections, there was no PD-1 staining in cancer cells, therefore a comparison between TMA and large sections results as to cancer cell expression was not feasible for PD-1.

### Quantitative reverse transcription PCR (qRT-PCR)

RNA was isolated from FFPE tissue sections using a fully automated isolation method of total RNA based on silica-coated magnetic beads (Versant Tissue Preparation Reagents Kit, Siemens Health Care, Erlangen, Germany) in combination with a liquid handling robot (Versant, Hamilton Robotics, Inc., Reno, NV, USA). Two 5-μm thick sections were cut from each paraffin block and transferred to a 1.5-ml tube. All tumor samples included in the study contained at least 30% tumor tissue as evaluated by H&E staining (median tumor content 60%). To achieve this tumor content, manual microdissection of the tumor area was performed if necessary. Expression of PD-1, PD-L1 as well as the normalization gene RPL37A was assessed in triplicate using a ViiA™ 7 Real-Time PCR device (Life Technologies, Darmstadt, Germany). Sequences of primers and probes have been published earlier.[42] deltaCT values were calculated with the formula CT_gene of interest_ - CT_RPL37A_. To obtain values proportional to actual RNA amounts, 40 - deltaCT was calculated.

### Statistical evaluation

Statistical analyses were performed with IBM SPSS Statistics 22 (Armonk, NY, United States), and GraphPad Prism v.5 (La Jolla, CA, USA). Associations were tested by Spearman, Chi square, Kruskal-Wallis or Mann-Whitney test, as indicated. Survival analysis was performed using the Kaplan-Meier method or Cox regression. All p-values were calculated two-tailed and p values < 0.05 were considered as significant. For Cox regression using continuous TILs data, TILs density (CD3+, PD-1+ or PD-L1+ TILs/mm^2^) were logarithmized using the formula: log10(TILs density + 1). For the determination of cutoff points, the Cutoff Finder online tool was applied (molpath.charite.de/cutoff) [43].

### TCGA datasets analysis

Gene expression datasets (Affymetrix, Agilent, RNAseq) from TCGA [18] were downloaded (https://tcga-data.nci.nih.gov/tcga) and investigated for the prognostic impact of PD-1 and PD-L1 gene expression using the software package R. Inclusion criteria for the analysis were: serous histology, and a G2 to G4 grading.

## SUPPLEMENTARY MATERIAL FIGURES AND TABLES


