# Supplementary material for: Prognostic impact of programmed cell death-1 (PD-1) and PD-ligand 1 (PD-L1) expression in cancer cells and tumor-infiltrating lymphocytes in ovarian high grade serous carcinoma
Source: Oncotarget. 2015 Nov 29;7(2):1486–99. doi: 10.18632/oncotarget.6429 (PMC4811475; doi:10.18632/oncotarget.6429)
Supplement: Supplementary file 1 [file oncotarget-07-1486-s001.pdf]

Prognostic impact of programmed cell death-1 (PD-1) and PD-ligand 1 (PD-L1) expression on cancer cells and tumor-infiltrating lymphocytes in ovarian high grade serous carcinoma

Supplementary Material

A

Staining intensity	Score
Negative	0
Weak	1
Moderate	2
strong	3
**Percentage of stained cancer cells**	
0%	0
1-10%	1
11-50%	2
51-80%	3
81-100%	4

Immunoreactivity Score (IRS) = $\text{score}_{\text{intensity}} \times \text{score}_{\text{percentage}}$

B

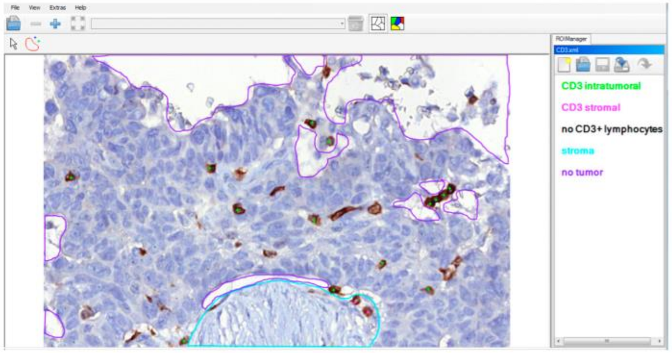

Suppl. Fig. S1 Illustration of PD-1 and PD-L1 immunohistochemical staining in cancer cells and TILs.: Multiplication of staining intensity score and score for the percentage of stained cells results in the immunoreactivity score (IRS), ranging from 0 to 12 **(A).** Screenshot from the ROI Manager showing the quantification of CD3+ TILs in an ovarian carcinoma microphotograph: stromal areas are labelled in turquoise, non-tissue areas in blue, intratumoral TILs in green, stromal TILs in pink **(B)**

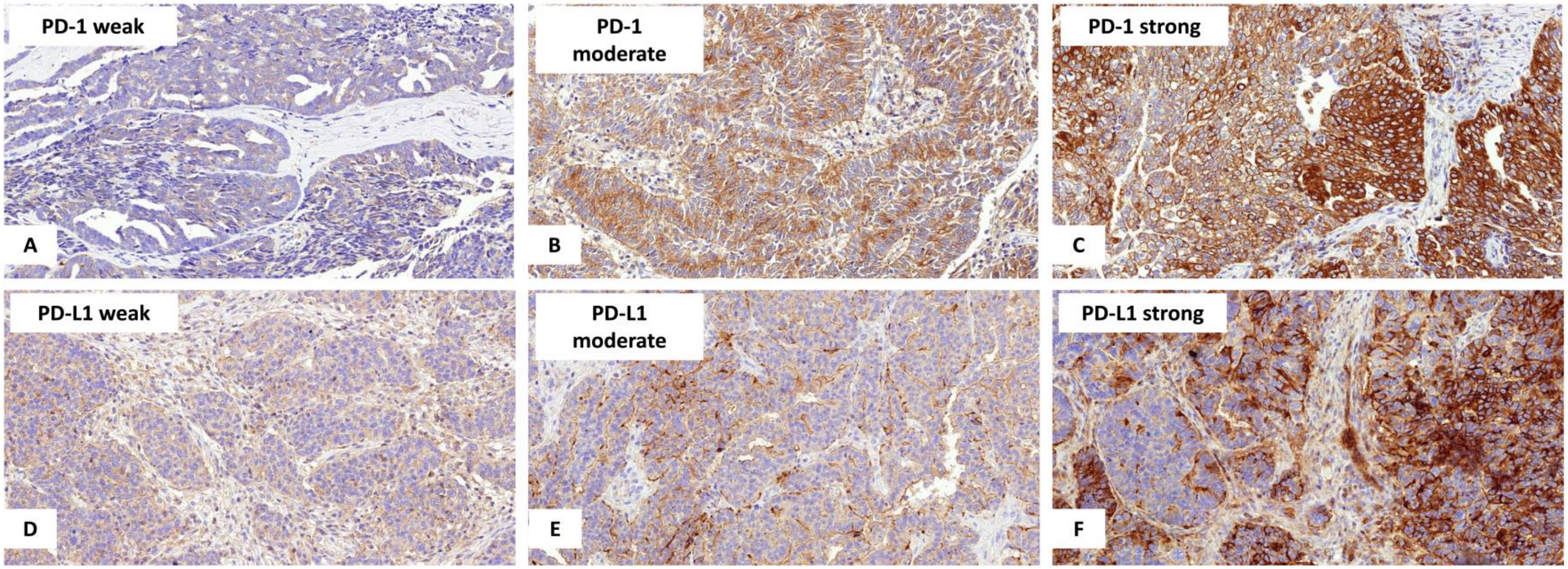

Suppl. Fig. S2 Examples for cancer cell immunohistochemical staining in varying intensities: PD-1 **(A-C)**, PD-L1 **(D-F)**

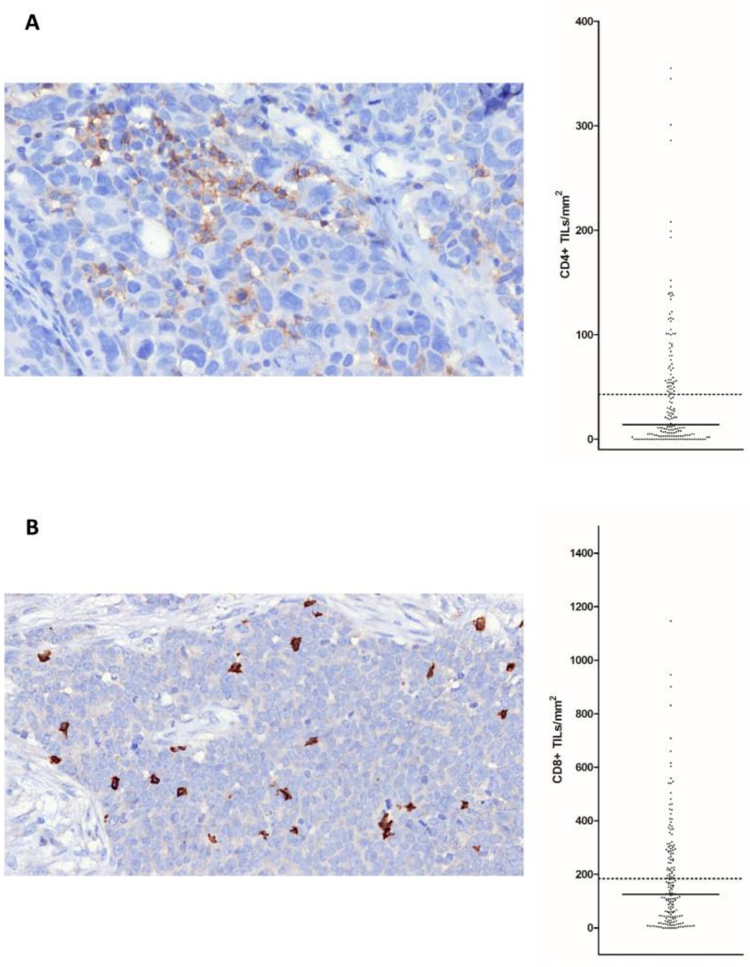

Suppl. Fig. S3 Distribution of CD4+ and CD8+ TILs: Immunohistochemical staining of CD4+ **(A)** and CD8+ **(B)** TILs. The diagrams on the right show the distribution of TILs/mm^2 tumor area (bars: median, dotted lines: means).

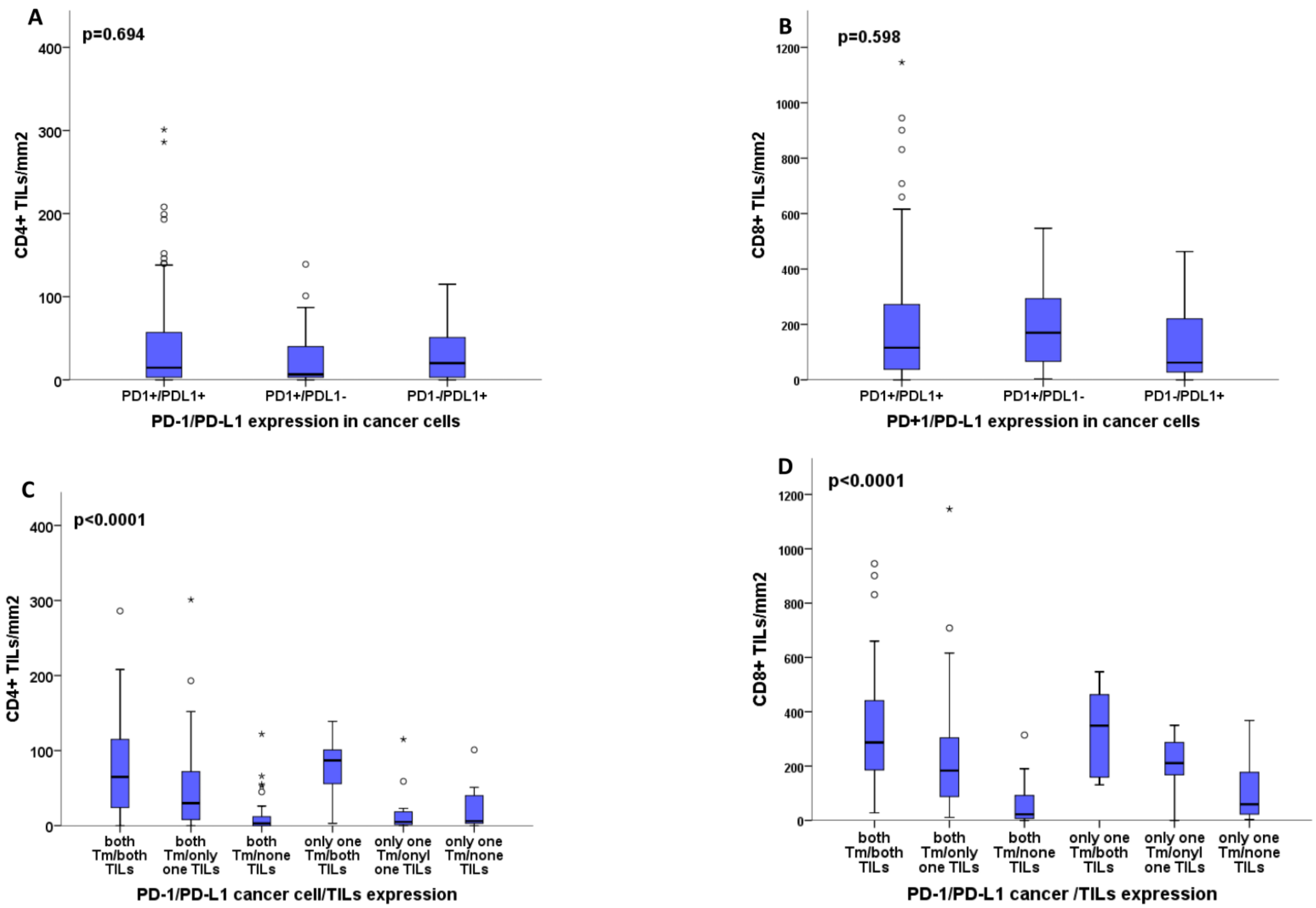

A
p=0.694
CD4+ TILs/mm2
400
300
200
100
0
PD1+/PDL1+
PD1+/PDL1-
PD1-/PDL1+
PD-1/PD-L1 expression in cancer cells
B
p=0.598
CD8+ TILs/mm2
1200
1000
800
600
400
200
0
PD1+/PDL1+
PD1+/PDL1-
PD1-/PDL1+
PD+1/PD-L1 expression in cancer cells
C
p<0.0001
CD4+ TILs/mm2
400
300
200
100
0
both Tm/both TILs
both Tm/only one TILs
both Tm/none TILs
only one Tm/both TILs
only one Tm/onyl one TILs
only one Tm/none TILs
PD-1/PD-L1 cancer cell/TILs expression
D
p<0.0001
CD8+ TILs/mm2
1200
1000
800
600
400
200
0
both Tm/both TILs
both Tm/only one TILs
both Tm/none TILs
only one Tm/both TILs
only one Tm/onyl one TILs
only one Tm/none TILs
PD-1/PD-L1 cancer /TILs expression

Suppl. Fig. 4 Interaction of CD4+ and CD8+ TILs with PD-1 and PD-L1 expression: Dependence of CD4+ **(A)** and CD8+ **(B)** TILs from combined PD-1 and PD-L1 expression in cancer cells. Dependence of CD4+ **(C)** and CD8+ **(D)** TILs from combined PD-1 and PD-L1 expression in cancer cells as well as in TILs.

(bars: median, box: interquartile range (IQR), whiskers: 1,5×IQR, dots and stars: outliers, p: Kruskal-Wallis test)

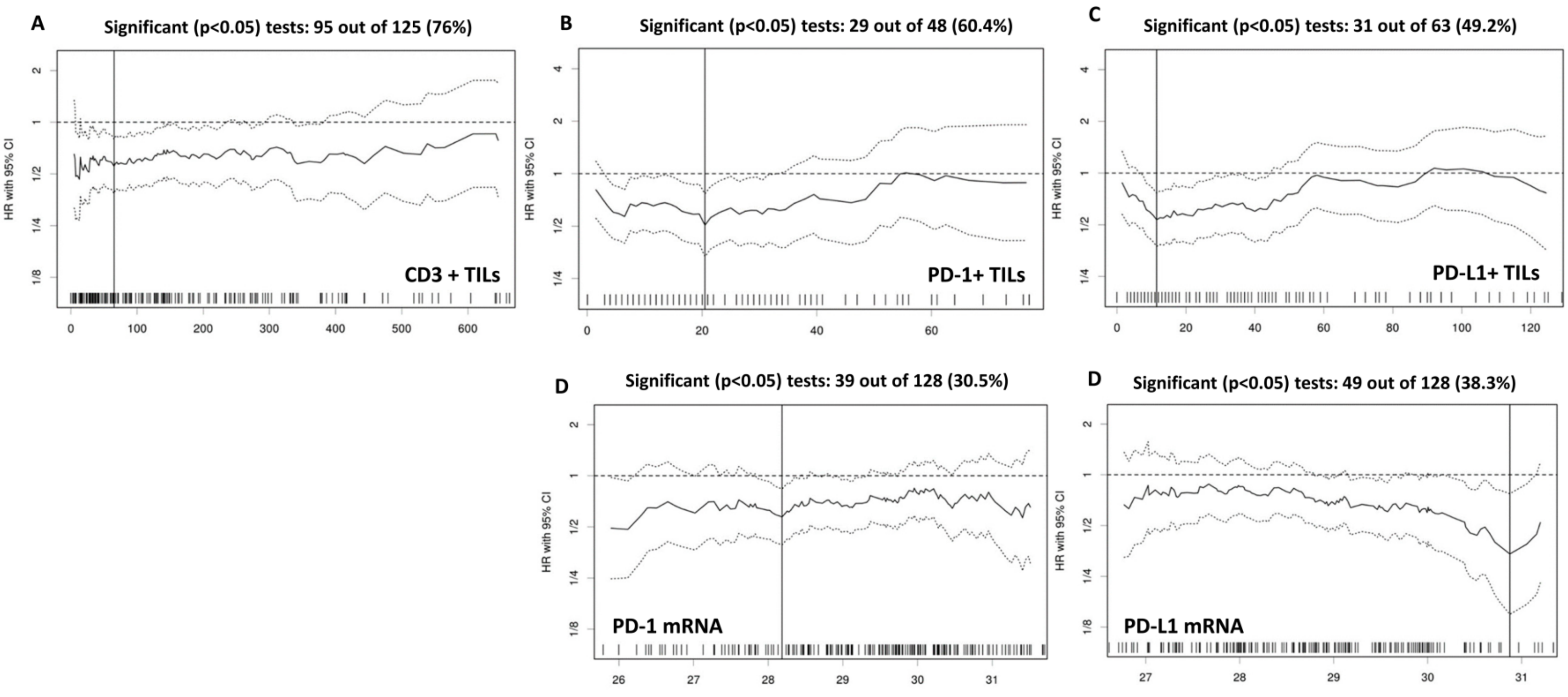

Suppl. Fig. S5 Cutoff point determination using Cutoff Finder (molpath.charite.de/cutoff): Hazard ratios (HR) are plotted against each possible cutoff point (CI: 95% confidence interval). Numbers and rates of significant cutoff points are indicated above each diagram: PFS according to CD3+ TILs **(A)**. PFS according to PD-1+ TILs **(B)**. PFS according to PD-L1+ TILs **(C)**. PFS according to PD-1 mRNA expression **(D)**. PFS according to PD-L1 mRNA expression **(E)**.

Supplemental Table S1: Survival Analysis - Overall survival

	univariate			*multivariate*	
	**no of events/ no of cases**	**median survival, months (SE)**	**p (log rank)**	**HR (95% CI)**	**p**
**PD-1 in tumor cells (membranous)**					
Negative	18/ 22	46.95 (8.7)		1	
positive	87/ 179	51.45 (9.3)	0.059	0.79 (0.46-1.37)	0.400
**PD-L1 in tumor cells (membranous)**					
Negative	18/ 24	37.91 (3.9)		1	
positive	88/ 178	50.63 (8.3)	0.045	0.51 (0.28-0.91)	0.023
**PD-1/PD-L1 in tumor cells combination**					
PD-1+/PD-L1+	74/ 159	65.68 (10.6)		1	
PD-1+/PD-L1-	15/ 21	37.91 (3.6)		2.28 (1.23-4.33)	
PD-1-/PD-L1+	14/ 18	47.74 (1.5)		1.18 (0.64-2.17)	
PD-1-/PD-L1-	2/ 2	23.62 (-)	0.051	3.07 (0.40-23.31)	0.065
**CD3+ TILs/mm^2**					
<=65	43/ 72	40.05 (2.7)		1	
> 65	61/ 128	66.83 (9.0)	0.003	0.63 (0.40-1.00)	0.037
**PD-1+ TILs/mm^2**					
<=11	57/ 97	42.51 (3.6)		1	
>11	47/ 103	68.37 (9.4)	0.012	0.58 (0.37-0.91)	0.018
**PD-L1+ TILs/mm^2**					
<=20	81/ 140	44.45 (2.77)		1	
>20	23/ 60	96.10 (-)	0.011	0.73 (0.44-1.20)	0.214
**PD-1/CD3 TILs combination**					
CD3+ TILs low	43/72	40.05 (2.8)		1	
CD3+ TILs high/PD-1+ TILs low	20/32	47.74 (9.1)		1.02 [0.53-1.96)	
CD3+ TILs high/PD-1+ TILs high	41/96	71.23 (13.4)	0.004	0.54 (0.33-.087)	0.023
**PD-L1/CD3 TILs combination**					
CD3+ TILs low	43/72	40.05 (2.7)		1	
CD3+ TILs high/PD-L1+ TILs low	39/70	65.31 (11.0)	0.004	0.64 (0.39-1.06)	
CD3+ TILs high/PD-L1+ TILs high	22/58	Not reached		0.58 (0.32-1.03)	0.106
**PD-1 mRNA (40 – deltaCT)**					
<=28.18	33/51	42.19 (4.6)		1	
>28.18	70/149	65.31 (10.9)	0.036	0.65 (0.40-0.98)	0.042
**PD-L1 mRNA (40 – deltaCT)**					
<=29.99	92/166	46.49 (3.3)		1	
>29.99	14/37	Not reached	0.045	0.55 (0.30-0.99)	0.047
**PD-1/PD-L1 mRNA combination**					

PD-1+/PD-L1+	13/36	Not reached		1	
PD1+ or PD-L1+	58/114	50.63 (11.0)		1.69 (0.89-3.21)	
PD-1-/PD-L1-	31/49	42.86 (4.4)	0.045	2.31 (1.16-4.61)	0.056

SE: standard error; HR: hazard ratio; CI: confidence interval; for each marker multivariate analysis was performed including age (<=60 vs > 60 years), stage (FIGO I/II vs III/IV), and residual tumor (none vs any)

Supplemental Table S2: Summary: Progression-Free Survival in Subgroup with Platinum-Based Chemotherapy

	univariate			*multivariate*	
	**no of events/ no of cases**	**median survival, months**	**p (log rank)**	**HR**	**p**
**PD-1 in tumor cells (membranous)**					
Negative	18/19	14.46 (2.4)		1	
positive	86/124	25.53 (2.7)	0.007	0.57	0.035
**PD-L1 in tumor cells (membranous)**					
Negative	18/19	14.13 (1.6)		1	
positive	87/125	25.69 (2.6)	0.002	0.41	0.001
**PD-1/PD-L1 in tumor cells combination**					
PD-1+/PD-L1+	72/109	26.88 (3.2)		1	
PD-1+/PD-L1-	16/17	14.13 (5.1)		2.68	
PD-1-/PD-L1+	15/16	15.11 (3.9)		1.78	
PD-1-/PD-L1-	1/1	15.31 (-)	0.002	2.23	0.004
**CD3+ TILs/mm^2**				1	
<=65	42/49	16.46 (2.3)		0.63	0.031
> 65	61/93	28.88 (2.8)	0.005		
**PD-1+ TILs/mm^2**				1	
<=11	55/66	16.46 (2.2)		0.63	0.005
>11	48/76	31.11 4.9)	0.002		
**PD-L1+ TILs/mm^2**				1	
<=20	80/103	20.24 (2.0)		0.72	0.055
>20	23/39	33.05 (4.8)	0.008		
**PD-1/CD3 TILs combination**				1	
CD3+ TILs low	42/49	16.46 (2.3)		0.72	
CD3+ TILs high/PD-1+ TILs low	16/21	17.41 (5.0)		0.53	0.031
CD3+ TILs high/PD-1+ TILs high	45/72	31.11 (5.1)	0.006		
**PD-L1/CD3 TILs combination**				1	
CD3+ TILs low	42/49	16.46 (2.3)		0.71	
CD3+ TILs high/PD-L1+ TILs low	39/56	24.74 (5.0)	0.006	0.61	0.064
CD3+ TILs high/PD-L1+ TILs high	22/37	33.05 (4.9)			
**PD-1 mRNA (40 – deltaCT)**					
<=28.18	35/38	15.11 (2.1)		1	
>28.18	67/104	27.57 (2.8)	0.002	0.46	0.001
**PD-L1 mRNA (40 – deltaCT)**					
<=30.45	87/116	20.70 (2.5)		1	
>30.45	17/28	35.81 (9.2)	0.020	0.41	0.002
**PD-1/PD-L1 mRNA combination**					

PD1+/PD-L1+	16/27	35.81 (7.5)		1	
PD1+ or PD-L1+	52/78	26.12 (3.9)		2.07	
PD1-/PD-L1-	33/36	15.11 (2.0)	0.003	3.69	<0.0001

SE: standard error; HR: hazard ratio; CI: confidence interval; for each marker multivariate analysis was performed including age (<=60 vs > 60 years), stage (FIGO I/II vs III/IV), and residual tumor (none vs any)

Supplemental Table S3: Summary: Progression-Free Survival in Subgroup with Platinum-Based Chemotherapy and no Residual Tumor

	univariate			***multivariate***	
	**no of events/ no of cases**	**median survival, months**	**p (log rank)**	**HR**	**p**
**PD-1 in tumor cells (membranous)**					
Negative	10/11	12.5 (2.8)		1	
positive	57/77	26.9 (3.4)	0.010	0.48	0.040
**PD-L1 in tumor cells (membranous)**					
Negative	12/12	13.4 (0.9)		1	
positive	56/77	27.6 (2.3)	0.001	0.30	<0.0001
**PD-1/PD-L1 in tumor cells combination**					
PD-1+/PD-L1+	48/68	27.57 (2.8)		1	
PD-1+/PD-L1-	11/11	14.13 (4.8)		3.4	
PD-1-/PD-L1+	8/9	14.46 (2.9)		1.9	
PD-1-/PD-L1-	0	-	0.001	-	0.001
**CD3+ TILs/mm^2**					
<=65	24/27	20.24 (3.5)		1	
> 65	42/60	29.18 (3.0)	0.005	0.53	0.018
**PD-1+ TILs/mm^2**					
<=11	33/38	17.41 (2.0)		1	
>11	33/49	31.11 (3.0)	0.002	0.41	0.001
**PD-L1+ TILs/mm^2**					
<=20	49/60	20.70 (3.4)		1	
>20	17/27	31.28 (3.0)	0.032	0.56	0.061
**PD-1/CD3 TILs combination**					
CD3+ TILs low	24/27	20.24 (3.5)		1	
CD3+ TILs high/PD-1+ TILs low	11/14	17.41 (4.2)		0.85	
CD3+ TILs high/PD-1+ TILs high	31/46	31.11 (5.9)	0.009	0.47	0.025
**PD-L1/CD3 TILs combination**					
CD3+ TILs low	24/27	20.24 (3.1)		1	
CD3+ TILs high/PD-L1+ TILs low	25/34	28.88 (7.1)		0.59	0.046
CD3+ TILs high/PD-L1+ TILs high	17/26	31.28 (4.68)	0.012	0.46	

**PD-1 mRNA (40 – deltaCT)**					
<=28.18	8/8	11.34 (2.4)		1	
>28.18	21/31	21.22 (2.2)	0.011	0.53	0.012
**PD-L1 mRNA (40 – deltaCT)**					
<=30.45	57/70	20.7 (3.3)		1	
>30.45	9/18	43.2 (3.0)	0.005	0.36	0.004
**PD-1/PD-L1 mRNA combination**					
PD1+/PD-L1+	8/17	38.74 (-)		1	
PD1+ or PD-L1+	33/44	27.43 (5.38)		2.6	
PD1-/PD-L1-	24/26	16.46 (3.61)	0.006	3.8	0.015

SE: standard error; HR: hazard ratio; CI: confidence interval; for each marker multivariate analysis was performed including age (<=60 vs > 60 years), and stage (FIGO I/II vs III/IV)
